# Clinical Implications of Plasma Galectin-3 in Heart Failure With Preserved Ejection Fraction: A Meta-Analysis

**DOI:** 10.3389/fcvm.2022.854501

**Published:** 2022-04-14

**Authors:** Yujiao Shi, Guoju Dong, Jiangang Liu, Xiong Shuang, Chunqiu Liu, Chenguang Yang, Wang Qing, Wenbo Qiao

**Affiliations:** ^1^Department of Post-graduate Institute, Chinese Academy of Traditional Chinese Medicine, Beijing, China; ^2^Department of Cardiovascular Internal Medicine, Xiyuan Hospital, Chinese Academy of Traditional Chinese Medicine, Beijing, China; ^3^National Clinical Research Center for Chinese Medicine Cardiology, Xiyuan Hospital, Chinese Academy of Traditional Chinese Medicine, Beijing, China

**Keywords:** Galectin-3, heart failure with preserved ejection fraction, diastolic heart failure, biomarkers, meta-analysis

## Abstract

**Background:**

Heart failure with preserved ejection fraction (HFpEF) is an increasing public health concern. Currently, data regarding the clinical application value of plasma Galectin-3 (Gal-3) in HFpEF are contradictory. Therefore, we performed the following meta-analysis to appraise the clinical implications of serum Gal-3 in HFpEF, including its capacity to predict new-onset disease, long-term unfavorable endpoints, and the degree of cardiac structural abnormality and left ventricular diastolic dysfunction (LVDD).

**Methods:**

PubMed, Embase, Scopus, and Web of Science were retrieved exhaustively from their inception until November 30, 2021, to obtain studies assessing the correlation between plasma Gal-3 and the clinical features of HFpEF (new-onset HFpEF, adverse outcomes, and echocardiographic parameters related to abnormal cardiac structure and LVDD).

**Results:**

A total of 24 papers containing 27 studies were ultimately included in the present research. The results of the meta-analysis revealed that high plasma Gal-3 levels are strongly associated with the following clinical characteristics of HFpEF: (i) the increased risk of new-onset HFpEF (HR: 1.11; 95% CI: 1.04-1.19; *p* = 0.910, I^2^ = 0%; *P* = 0.002); (ii) the high risk of adverse outcomes of HFpEF patients [all-cause death (HR: 1.55; 95% CI: 1.27-1.87; *p* = 0.138, I^2^ = 42%; *P* = 0.000) and the composite events [all-cause death and HF hospitalization (HR: 1.50; 95% CI: 1.30-1.74; *p* = 0.001, I^2^ = 61%; *P* = 0.000) or cardiovascular (CV) death and HF hospitalization (HR: 1.71; 95% CI: 1.51-1.94; *p* = 0.036, I^2^ = 58%; *P* = 0.000)]; (iii) echocardiographic indices [E/e ratio (r: 0.425, 95% CI: 0.184-0.617; *p* = 0.000, I^2^ = 93%; *P* = 0.001) and DT (r: 0.502, 95% CI: 0.061-0.779; *p* = 0.001 I^2^ = 91%; *P* = 0.027)].

**Conclusions:**

Plasma Gal-3 might be employed as an additional predictor for new-onset HFpEF, the adverse prognosis in HFpEF patients (all-cause death, the composite endpoints of all-cause death and HF hospitalization or CV death and HF hospitalization), and the severity of LVDD in HFpEF populations.

## Introduction

Heart failure (HF) with preserved ejection fraction (HFpEF) is a heterogeneous medical condition not only characterized by a normal left ventricular ejection fraction (LVEF) and a slew of symptoms and signs due to either cardiac remodeling or left ventricular diastolic dysfunction (LVDD) (1), but also accompanied by other extra-cardiac syndromes such as renal dysfunction (2), skeletal muscle dysfunction (3). It has become a significant public health challenge, affecting around half of all HF populations worldwide (4), with 5-year mortality and rehospitalization following similar patterns as HF with reduced ejection fraction (HFrEF) (5). Furthermore, the pathophysiological underpinnings of HFpEF remain poorly understood (6), and there are limitations in clinical diagnosis and medical therapy (7). In this context, making an early prediction of the first occurrence of the disease or prognosis is crucial to facilitate the employment of timely interventions to slow or halt disease progression and improve long-term consequences.

Cardiac biomarkers are positively associated with the pathophysiology of HF, allowing them to be applied to track disease severity and progression and determine appropriate therapy (8). Currently, two clinically accessible makers, high sensitivity troponin T and N-terminal pro-B-type natriuretic peptide (NT-proBNP), contribute significantly to the diagnosis and prognosis of HF (9–12). Unfortunately, unlike in HFrEF, the value of these two markers in individuals with HFpEF may be restricted (13). Therefore, exploring new biomarkers with clinical value has gained increased attention. Galectin-3 (Gal-3), a new promising candidate marker with additional diagnostic and prognostic value in HF, is implicated in inflammation and myocardial fibrosis development (14, 15). In recent years, multiple clinical trials have analyzed the connection between plasma Gal-3 and new-onset HFpEF, unfavorable prognosis of HFpEF patients, and the severity of cardiac structural and functional alterations of HFpEF populations. Some trials have shown a correlation between plasma Gal-3 and HFpEF, while others have not. Accordingly, we implemented a meta-analysis to estimate the clinical application value of plasma Gal-3 in HFpEF, including its capacity to predict new-onset disease, long-term unfavorable outcomes, and the degree of cardiac structural abnormality and LVDD.

## Materials and Methods

### Literature Search Strategy

A meta-analysis was undertaken based on Preferred Reporting Items for Systematic Reviews and Meta-Analysis (PRISMA) (16). Two selected participants (Shi and Xiong) systematically conducted documentary searches in four online databanks (PubMed, Embase, Scopus, and Web of Science). We searched for literature in English published from the inception of each database until November 30, 2021. Terms related to “Heart Failure, Diastolic,” “Heart Failure with Preserved Ejection Fraction,” “Diastolic Dysfunction,” “Preserved Ejection Fraction,” “Galectin-3,” “Gal-3,” and “Biomarker” were utilized depending on the retrieval regulation of each database. For PubMed, the following search was executed: (Heart Failure, Diastolic [MeSH Terms]) OR (Heart Failure with Preserved Ejection Fraction [Title/Abstract]) OR (Diastolic Dysfunction [Title/Abstract]) OR (Preserved Ejection Fraction [Title/Abstract]) AND (Galectin-3 [MeSH Terms]) OR (Gal-3 [Title/Abstract]) OR (Biomarker [Title/Abstract]).

### Literature Inclusion and Exclusion Criteria

During the screening process, candidate research must fulfill the following inclusion criteria: (i) study design: observational studies were included (prospective and retrospective cohort studies, case-control studies, *post hoc* analysis studies); (ii) endpoints: The connection between plasma Gal-3 concentrations and the risk of new-onset HFpEF or adverse endpoints of HFpEF patients [all-cause death, cardiovascular (CV) death, and HF hospitalization], with the results presented as hazard ratios (HRs) and 95% confidence intervals (CIs). Moreover, the relevance of plasma Gal-3 concentrations and echocardiographic parameters [the ratio of peak early (E) and late (A) diastolic velocities of LV inflow, the ratio of E and medial mitral annular velocity (e), LV mass index (LVMI), left atrial (LA) volume index (LAVI), and E-wave deceleration time (DT)], and the findings were expressed as correlation coefficient (r). Articles will be eliminated if they satisfy at least one of the following exclusion criteria: (i) irrelevant articles or duplicated studies; (ii) the papers were case reports, reviews, letters, commentaries, editorials, or non-human studies; (iii) the articles reported irrelevant endpoints; (iv) the articles lacked full text or the sufficient crude data.

### Literature Quality Evaluation Criteria

The quality estimation of the enrolled studies was evaluated by two independent reviewers (Liu and Yang) using the Newcastle–Ottawa Quality Assessment Scale (NOS) system, a “star-based” grading system which is comprised of three parts (selection, comparability, and outcomes). Specifically, we assessed the following characteristics of each study: (i) representativeness of the exposed cohort, (ii) selection of the non-exposed cohort, (iii) ascertainment of exposure, (iv) demonstration that outcome of interest was not present at the start of the study, (v) comparability of cohorts based on study design or analysis, (vi) assessment of outcomes, (vii) was follow-up long enough for outcomes to occur, and (viii) adequacy of follow-up of cohorts.

### Data Extraction

The required data from the included research were extracted and tabulated in specifically constructed Microsoft Excel spreadsheets for analysis. The extracted contents were as follows: (i) publication details: last name of the first author, year of publication, and the country setting; (ii) demographic characteristics: sample size, proportions of males, and mean age; (iii) study details: study design, mean or median concentrations of plasma Gal-3, variables adjusted, follow-up duration, and endpoints; (iv) NOS quality score. Two independent researchers (Wang and Qiao) conducted data extraction, and disagreements were resolved by mutual coordination or third-party adjudication (Dong and Liu).

### Statistical Analysis

STATA (Version 16.0) was used to analyze the association of plasma Gal-3 and the risk of new-onset HFpEF or adverse outcomes. The connection between plasma Gal-3 and echocardiographic data was analyzed using Comprehensive Meta-Analysis Software (Version 3.0). The heterogeneity among the included studies was appraised by the Cochran Q statistics (chi-square test) and quantified with the I^2^ statistic. The fixed-effect model was employed when the *Q*-test (I^2^ < 50%, *p* > 0.05) revealed no significant heterogeneity across studies. When the *Q*-test (I^2^ > 50% or *p* < 0.05) found prominent heterogeneity among studies, the random-effect model was utilized, followed by a Galbraith plot to explore the source of heterogeneity if over 10 trials were involved. Furthermore, the Funnel plot, Begg's test, and Egger's test were conducted to estimate publication bias, and sensitivity analysis was employed to evaluate the impact of single research on the overall estimate by omitting one study each time.

## Results

### Literature Search Results

**Figure 1:**
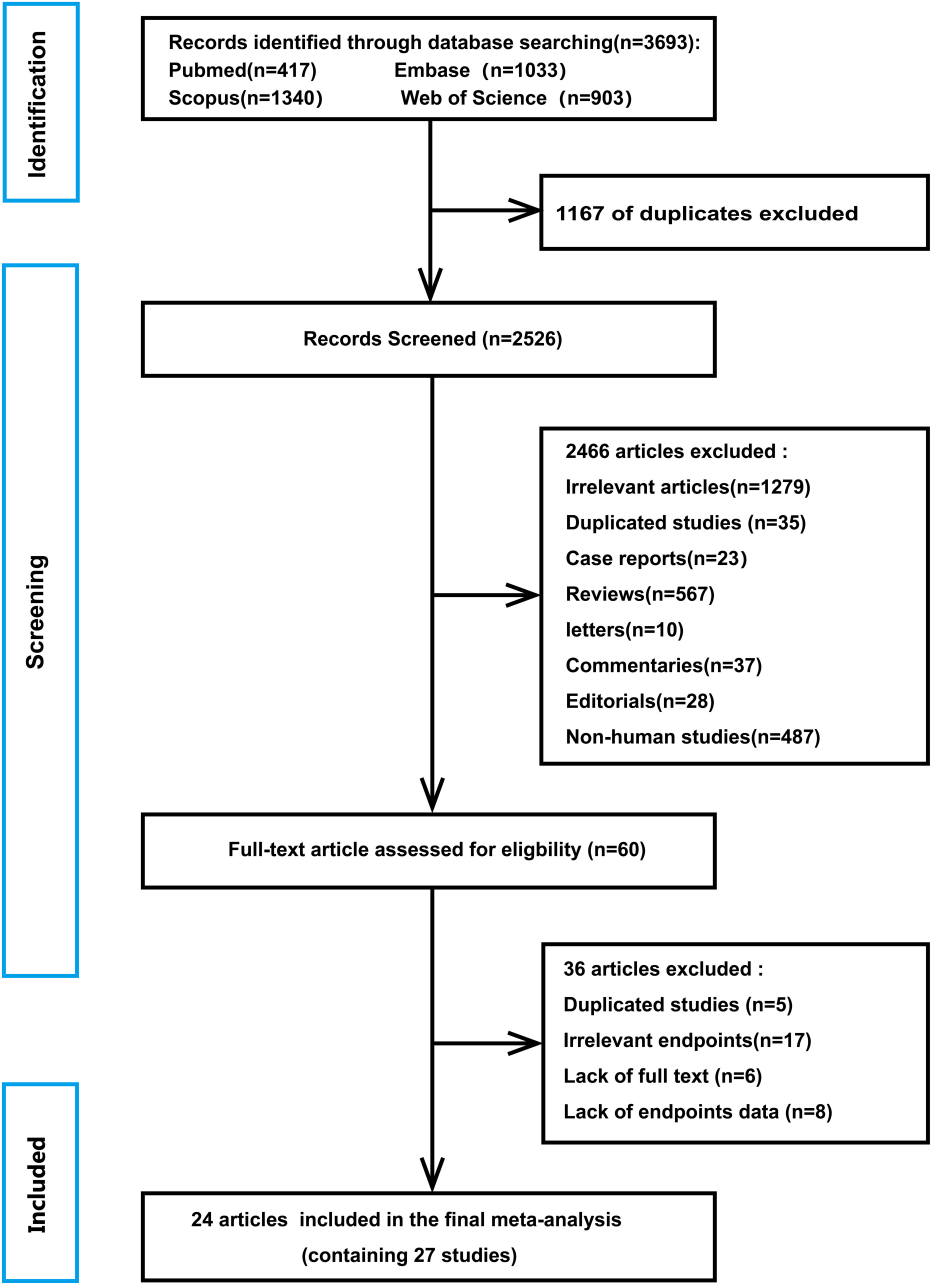
The procedure of database search and text screening.

A flowchart of database search and text screening procedure is demonstrated in Figure 1. A total of 3,693 publications were retrieved through database searching, consisting of 417 from PubMed, 1,033 from Embase, 1,340 from Scopus, and 903 from Web of Science. After excluding 1,167 duplicate literature, we screened the titles and abstracts of the 2,526 papers. Then, 2,466 articles were removed following the inclusion and exclusion criteria. Finally, among the remaining 60 studies, two independent researchers read the full text and excluded 36 records due to repetitive research, irrelevant results, and insufficient data. Overall, 24 articles (17–40) in total were enrolled in the meta-analysis.

### Characteristics of Included Literature

A total of 24 articles published from 2011 to 2021 were investigated. These papers contained 27 studies, including 15 prospective cohort studies, 10 case-control studies, and two *post hoc* analysis studies. Table 1 shows the baseline characteristics of the selected research. A global population of 31,052 patients was involved, and the follow-up period ranged from 4 to 144 months. Except for the study conducted by Beltrami et al., which lacked relevant data, additional 26 studies included 16,299 males with an average age between 49 and 73 years. Eighteen studies detected serum Gal-3 using enzyme-linked immunosorbent assay (ELISA), while the other nine studies utilized enzyme-linked fluorescence assay (ELFA), multiplex immunoassay, luminex® Bead-Based multiplexed assay et al. respectively. Additionally, most studies adjusted for other variables possibly affecting the association of plasma Gal-3 and research outcomes, including age, sex, race, history of CV disease, medication history, laboratory tests et al. The NOS scores of included research varied from 6 to 9, indicating that the methodological quality was generally reliable (Supplementary Table 1).

**Table 1:** Baseline characteristics of the 27 selected research.

**References**	**Country**	**Sample size, ***n*****	**Males,** ***n***	**Age, mean (SD), years**	**Study design**	**Gal-3, mean (SD) or median, ng/ml**	**Analysis method**	**Univariate(U)/ multivariate (M)**	**Variables adjusted**	**Follow-up** **Duration (months)**	**Endpoint(s)**	**NOS quality score**
de Boer et al. (17) (CHS)	US	5,277	2,239	73	Prospective cohort	15.2	ELISA	M	Age, sex, race, body mass index (BMI), smoking status, diabetes, LV hypertrophy, systolic blood pressure, left bundle branch block, hypertension treatment.	144	New-onset HFpEF	9
de Boer et al. (17) (FHS)	US	3,431	1,605	59	Prospective cohort	13.7	ELISA	M	Age, sex, race, BMI, smoking status, diabetes, LV hypertrophy, systolic blood pressure, left bundle branch block, hypertension treatment.	144	New-onset HFpEF	9
Bansal et al. (18)	US	3,314	1,794	57.5	Prospective cohort	15.7	ELISA	M	Age, sex, race, BMI, smoking status, diabetes, cardiovascular disease, systolic blood pressure, the use of (β-blockers, phosphate, angiotensin-converting enzyme inhibitor (ACEI), aldosterone receptor blockers), 24 h urinary protein, glomerular filtration rate (e GFR), parathyroid hormone, fibroblast growth factor-23.	94.8	New-onset HFpEF	8
de Boer et al. (19)(PREVEND)		8,322	4,161	49	Prospective cohort	12.6	ELISA	M	—	120	New-onset HFpEF	8
Beltrami et al. (20)	Italy	98	—	—	Case-control	20	ELISA	M	Renal dysfunction.	6	All-cause death and HF hospitalization, echocardiographic data (E/A, E/e)	7
Moliner et al. (21)	Spain	1,069	786	66.2	Case-control	19.2	ELFA	U	—	79.2	All-cause death and HF hospitalization, CV death and HF hospitalization	7
Tromp et al. (22)	Netherlands	460	304	70.6	Case-control	19.3	ELISA	M	Age, sex, history of myocardial infarction, atrial fibrillation, anemia, diabetes, systolic blood pressure, e GFR.	18	All-cause death and HF hospitalization	8
Meijers et al. (23) (PRIDE)	Netherlands	181	84	72.9	Prospective cohort	14.9	ELISA	M	Age, gender, New York Heart Association functional (NYHA) class, BNP, LVEF, e GFR.	4	All-cause death and HF hospitalization	8
Meijers et al. (23) (UMD H-23258)	Netherlands	592	365	70.8	Prospective cohort	19.8	ELISA	M	Age, gender, NYHA class, BNP, LVEF, e GFR.	4	All-cause death and HF hospitalization	8
Edelmann et al. (24)	Germany	415	198	67	*Post hoc* analysis studies	12.5	ELISA	M	Age, sex, atrial fibrillation, mean arterial pressure, estimated glomerular filtration rate, hemoglobin, NT-pro BNP.	12	All-cause death and HF hospitalization, echocardiographic data (E/e, LAVI, LVMI)	9
Hage et al. (25)	Sweden	86	42	73	Prospective cohort	—	Multiplex immunoassay	M	Age, sex, NT-pro BNP.	19.3	All-cause death and HF hospitalization, echocardiographic data (E/e, LAVI, LVMI)	7
Chirinos et al. (26)	US	379	203	70	Prospective cohort	—	Luminex® Bead-Based multiplexed assay	M	Age, sex, BMI, systolic blood pressure, smoking status, diabetes, chronic obstructive pulmonary disease, HF duration > 18 months, the use of (β-blockers, ACEI), LVEF, NYHA class, creatinine.	33.4	All-cause death and HF hospitalization	8
Kanagala et al. (27)	US	130	65	72.5	Case-control	—	ELISA	M	Age, gender, diabetes, BNP, e GFR.	47.6	All-cause death and HF hospitalization	8
de Boer et al. (28)	US	592	384	72	Prospective cohort	20.0	ELISA	M	Age, gender, diabetes, BNP, e GFR, LVEF.	18	All-cause death and HF hospitalization	8
Carrasco-Sánchez et al. (29)	Spain	419	254	76.2	Prospective cohort	13.8	ELISA	M	Age, diabetes, anemia, NYHA class, NT-pro BNP, urea, e GFR, serum sodium.	12	All-cause death and HF hospitalization	7
French et al. (30)	US	1,385	921	57	Prospective cohort	17.6	ELISA	M	Age, sex, race, cardiomyopathy etiology, cardiac resynchronization therapy, defibrillator use, creatinine	55.2	All-cause death and HF hospitalization	7
van der Velde et al. (31) (CORONA)	Netherlands	1,329	1,024	71.6	Prospective cohort	20.1	BG Medicine Gal-3 Assay	M	Age, sex, diabetes, LVEF, e GFR, NT-pro BNP.	6	All-cause death and HF hospitalization, CV death and HF hospitalization, all-cause death	8
van der Velde et al. (31) (COACH)	Netherlands	324	195	69.9	Prospective cohort	18.2	ELISA	M	Age, sex, diabetes, LVEF, e GFR, NT-pro BNP.	6	All-cause death and HF hospitalization, all-cause death	8
Yamamoto et al. (32)	Japan	616	377	74	Prospective cohort	8.45	ELISA	M	Age, sex, BMI, history of HF hospitalization, chronic obstructive pulmonary disease, ischemic etiology, smoking status, the use of (β-blockers, ACEI), systolic blood pressure, LVEF, BNP, e GFR.	12	CV death and HF hospitalization, all-cause death	7
Cui et al. (33)	China	300	112	67	Case-control	9.42	Human Galectin-3 Assay Kit	M	Age, sex, hypertension, the use of (β-blockers, aldosterone receptor blockers), systolic blood pressure, diastolic blood pressure, NYHA class, LVEF, LDL cholesterol, e GFR.	12	CV death and HF hospitalization	8
De Marco et al. (34)	Canada	248	135	71.56	*Post hoc* analysis studies	20.0	—	M	Age, sex, strata, diabetes, treatment, and baseline biomarker values.	31.2	CV deaths and HF hospitalizations	8
Trippel et al. (35)	Germany	1,386	680	66.8	Prospective cohort	13.4	ELISA	M	—	120	All-cause death, CV deaths and HF hospitalizations	8
Mitic et al. (39)	US	77	58	61.81	Case-control	22.32	Quantikine^R^	—	—	—	Echocardiographic data (E/A)	7
Wu et al. (40)	Taiwan	176	67	67.74	Case-control	—	ELISA	—	—	—	Echocardiographic data (E/e, LVMI, DT)	6
Ansari et al. (37)	Germany	70	36	65	Case-control	—	The Gal-3 assay on an Architect i1000 analyzer	—	—	—	Echocardiographic data (E/A, E/e, DT)	7
Zile et al. (38)	US	294	166	71.25	Case-control	19.0	ELISA	—	—	—	Echocardiographic data (E/A, E/e)	7
Polat et al. (36)	Turkey	82	44	58.5	Case-control	—	—	—	—	—	Echocardiographic data (E/e, LAVI, LVMI)	6

### Meta-Analysis

#### Association of Elevated Plasma Gal-3 Levels With New-Onset HFpEF

**Figure 2:**
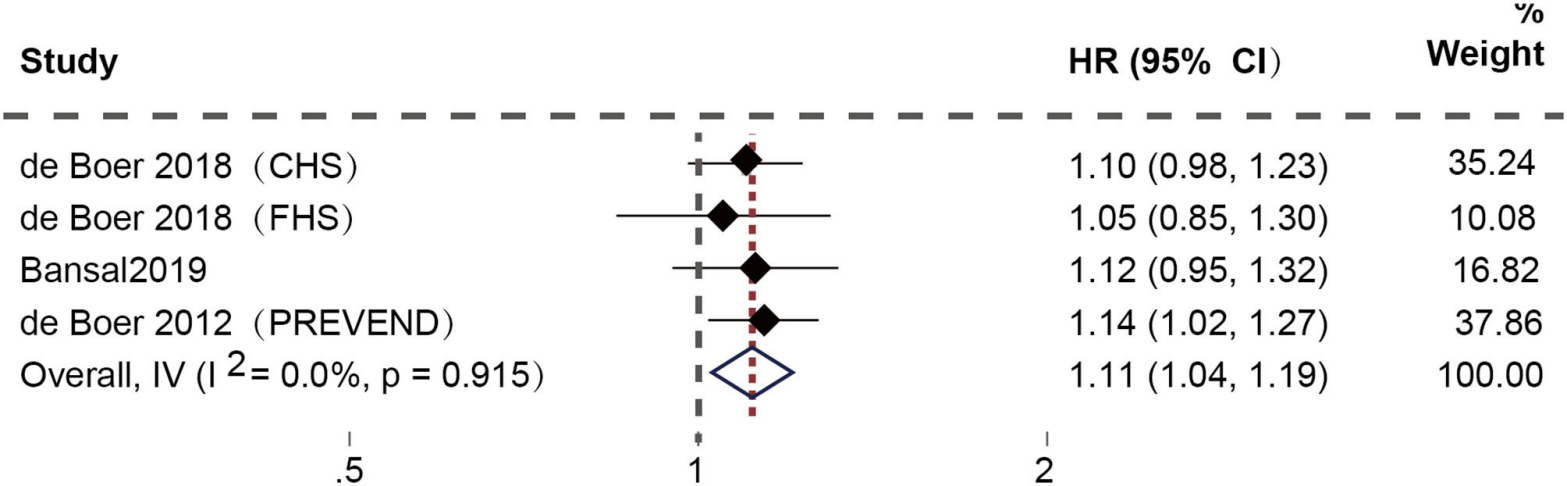
Association between elevated plasma Gal-3 levels and new-onset HFpEF.

After excluding duplicate studies, four studies (17–19) focused on the connection between high plasma Gal-3 levels and the risk of new-onset HFpEF. Among them, de Boer et al. conducted three prospective cohorts, including the Cardiovascular Health Study (CHS), the Framingham Heart Study (FHS), and the Prevention of Renal and Vascular End-stage Disease (PREVEND) cohort. The fixed-effect model was applied since the examination of heterogeneity showed no heterogeneity. The results demonstrated a substantial correlation between high plasma Gal-3 levels and a higher risk of new-onset HFpEF (HR: 1.11; 95% CI: 1.04-1.19; *p* = 0.910, I^2^ = 0%; *P* = 0.002) (Figure 2).

#### Association of Elevated Plasma Gal-3 Levels With Adverse Outcomes of HFpEF Patients

**Figure 3:**
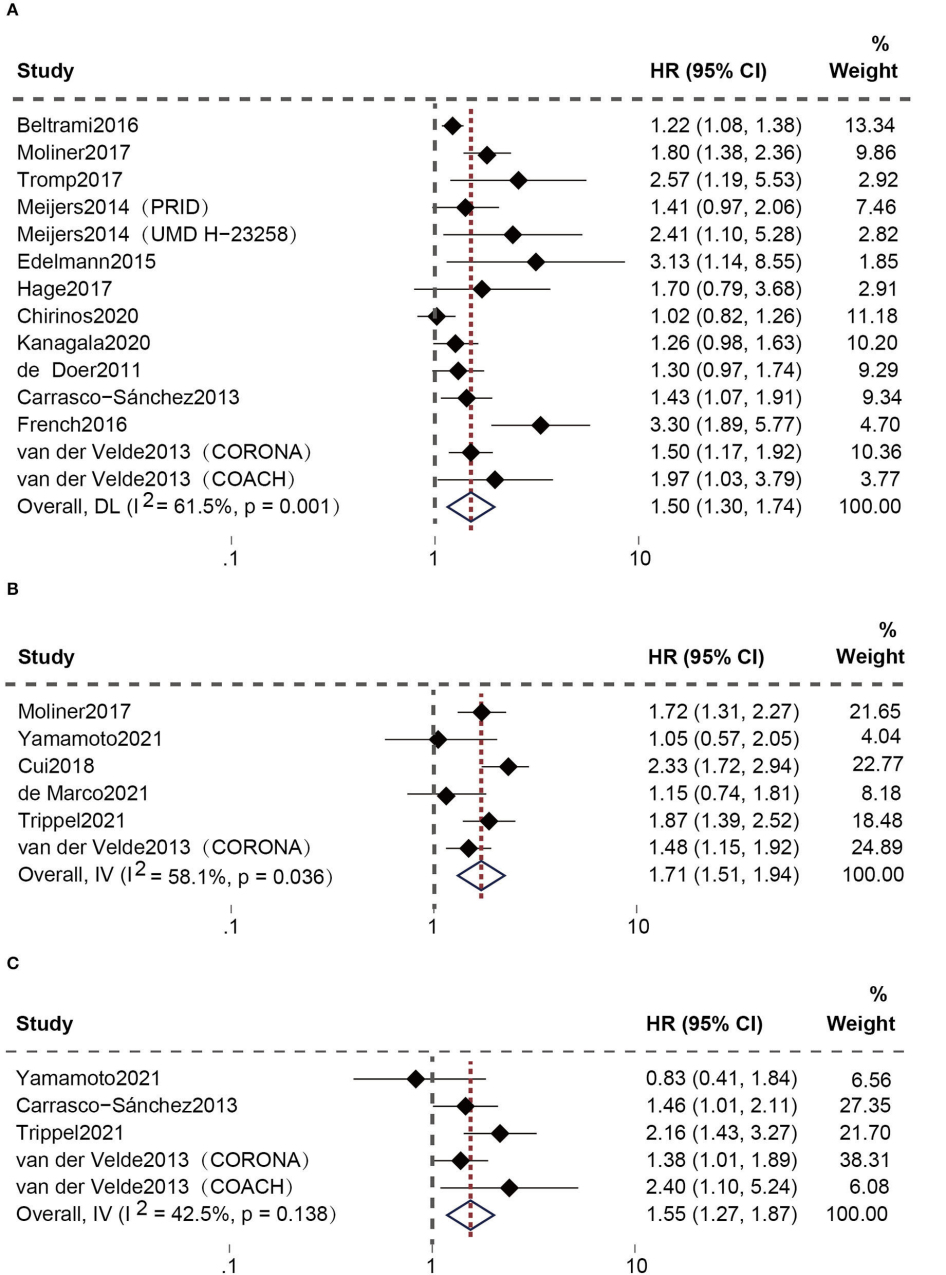
Association between elevated plasma Gal-3 levels and adverse outcomes of HFpEF patients: **(A)** Association between plasma Gal-3 and the composite endpoint of all-cause death and HF hospitalization. **(B)** Association between plasma Gal-3 and the composite endpoint of CV death and HF hospitalization. **(C)** Association between plasma Gal-3 and all-cause death.

After eliminating repeated cohorts, 21 studies (20–35) assessed the connection between high plasma Gal-3 levels and the composite adverse outcomes of HFpEF patients. Among these, Meijers et al. and van der Velde et al. performed two longitudinal cohorts separately [the Pro-BNP Investigation of Dyspnea in the Emergency Department (PRIDE), the University of Maryland Pro-BNP for Diagnosis and Prognosis in Patients Presenting with Dyspnea Study (UMD H-23258), the Controlled Rosuvastatin Multinational Trial in Heart Failure (CORONA), and the Coordinating study evaluating Outcomes of Advising and Counseling in Heart Failure (COACH)]. The random-effect model was utilized owing to the existence of apparent heterogeneity. Elevated plasma Gal-3 levels are connected to a greater risk of the composite outcomes [all-cause death and HF hospitalization (HR: 1.50; 95% CI: 1.30-1.74; *p* = 0.001, I^2^ = 61%; *P* = 0.000) (Figure 3A) or CV death and HF hospitalization (HR: 1.71; 95% CI: 1.51-1.94; *p* = 0.036, I^2^ = 58%; *P* = 0.000) (Figure 3B)]. Moreover, five studies (29, 31, 32, 35) specifically analyzed the correlation between increased plasma Gal-3 and the risk of all-cause death in HFpEF patients. The fixed-effect model was utilized since heterogeneity was insignificant. The results confirmed a substantial relationship between them (HR: 1.55; 95% CI: 1.27-1.87; *p* = 0.138, I^2^ = 42%; *P* = 0.000) (Figure 3C).

#### Association of Elevated Plasma Gal-3 Levels With Echocardiographic Parameters

**Figure 4:**
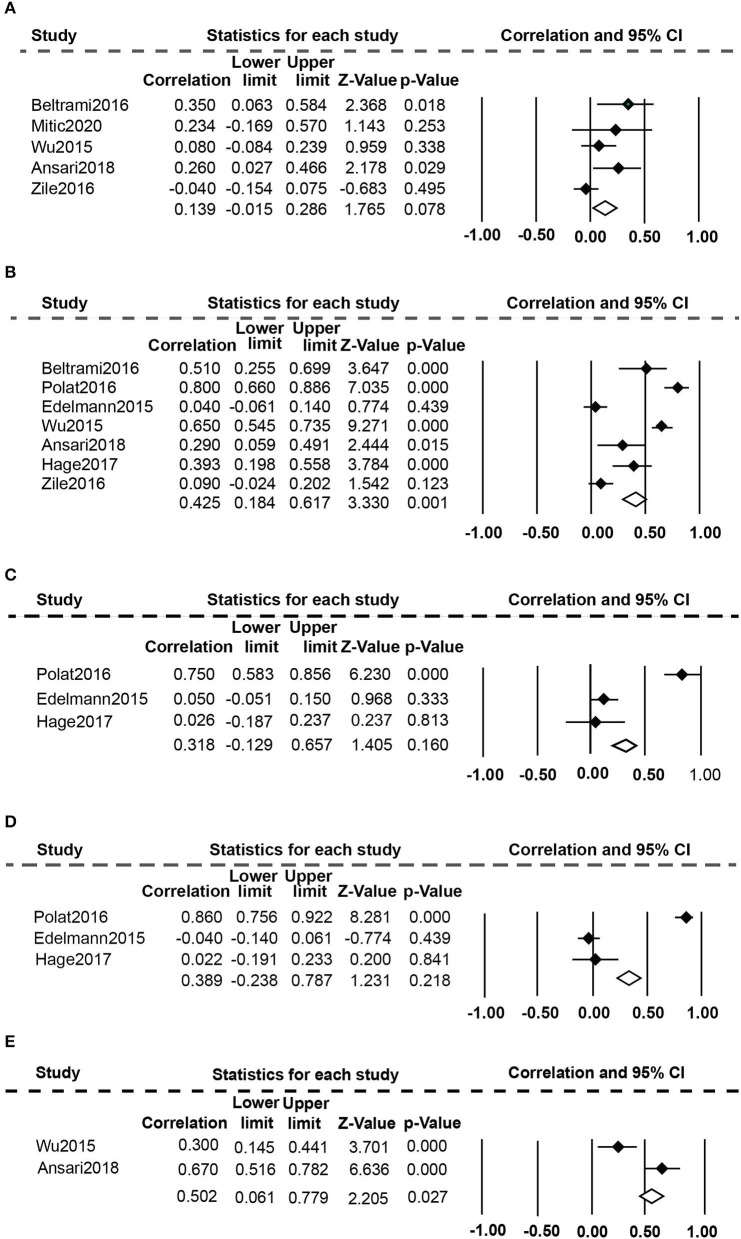
Association between elevated plasma Gal-3 levels and echocardiographic parameters: **(A)** Association between plasma Gal-3 and E/A. **(B)** Association between plasma Gal-3 and E/e. **(C)** Association between plasma Gal-3 and LAVI. **(D)** Association between plasma Gal-3 and LVMI. **(E)** Association between plasma Gal-3 and DT.

Eight studies (20, 24, 25, 36–40) evaluated the relationship between high plasma Gal-3 levels and echocardiographic data (Figure 4). The random-effect model was employed because of the presence of substantial heterogeneity. Elevated plasma Gal-3 levels are significantly related to E/e ratio (r: 0.425, 95% CI: 0.184-0.617; *p* = 0.000, I^2^ = 93%; *P* = 0.001) (Figure 4B) and DT (r: 0.502, 95% CI: 0.061-0.779; *p* = 0.001 I^2^ = 91%; *P* = 0.027) (Figure 4E), but not to E/A ratio (r: 0.139, 95% CI: −0.015-0.286; *p* = 0.034, I^2^ = 61%; *P* = 0.078) (Figure 4A), LAVI (r: 0.318, 95% CI: −0.129-0.675; *p* = 0.000, I^2^ = 93%; *P* = 0.160) (Figure 4C), and LVMI (r: 0.389, 95% CI: −0.238-0.787; *p* = 0.000, I^2^ = 96%, *P* = 0.218) (Figure 4D).

### Heterogeneity, Publication Bias, and Sensitivity Analyses

**Figure 5:**
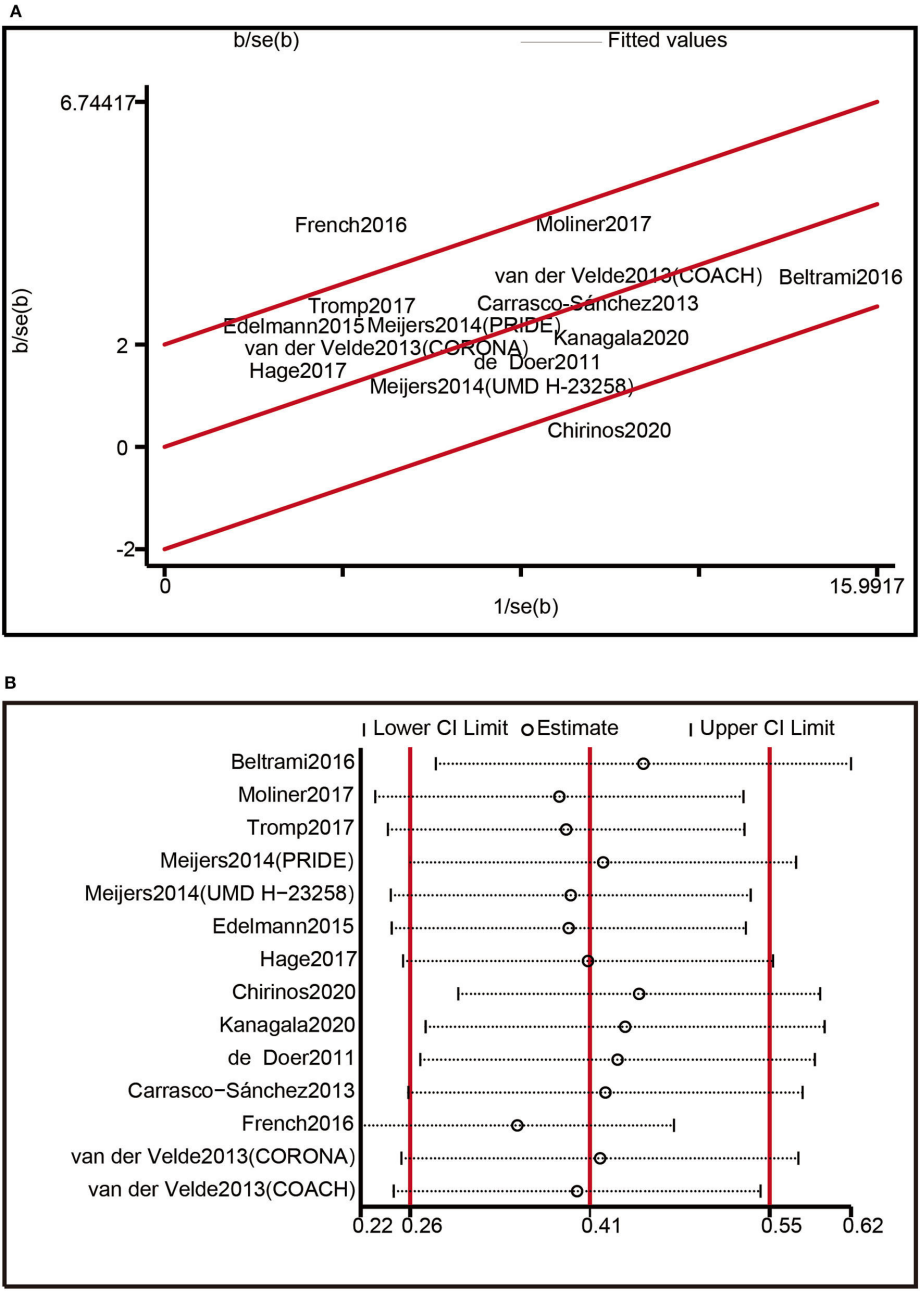
Heterogeneity analysis: **(A)** Heterogeneity was evaluated by Galbraith radial plot. **(B)** Sensitivity analysis.

Our findings on the composite outcomes of all-cause death and HF hospitalization, which included 14 trials, revealed significant heterogeneity. The Galbraith radial plot was applied to investigate the origin of variability. Two studies conducted by French et al. and Chirinos et al. were considered as probable sources of the heterogeneity (Figure 5A). The Funnel plot analysis was asymmetrical (Supplementary Figure 1), and the *p*-values of Begg's test (*p* = 0.029) (Supplementary Figure 2) and Egger's test (*p* = 0.002) (Supplementary Figure 3) were lower than 0.05, suggesting the existence of publication bias across included studies. Nevertheless, a sensitivity analysis removing one research each time indicated that none of the individual trials substantially impacted the pooled estimates (Figure 5B). Overall, the results of this study were reasonably constant.

## Discussion

HF represents the end-stage manifestation of numerous cardiovascular illnesses. The 2021 European Society of Cardiology (ESC) guidelines advocated categorizing HF into three groups based upon the measurement of LVEF: HFrEF (LVEF ≤ 40%), HF with mildly reduced ejection fraction (HFmrEF, LVEF 41-49%), and HFpEF (LVEF ≥ 50%) (41). HFpEF is becoming an overwhelming challenge for physicians as the prevalence of hypertension, diabetes, chronic kidney disease, and advancing age increases, accounting for approximately one-half of all HF (42, 43). Moreover, evidence-based therapies are finite, leading to a high prevalence of hospital admissions and mortality among the elderly population (44). Presently, HFpEF is diagnosed depending on representative symptoms and signs of HF (breathlessness, fatigue, edema, et al.), an LVEF of more than 50%, objective evidence of cardiac structural or functional abnormalities, and an increasing level of BNP or NT pro-BNP (45). However, most sufferers lack specific clinical manifestations at the initial stages and are usually detected only in the advanced phase of HFpEF (46). Exploring efficient biomarkers for identifying at-risk individuals, early diagnosis, prognosis, and treatment monitoring is therefore essential.

Gal-3, a soluble β-galactoside-binding lectin implicated in the process of cell proliferation, adhesion, migration, and apoptosis, appeared to promote inflammation or tissue fibrosis in HF (15), atherothrombosis (47), atrial fibrillation (48), and chronic kidney disease (49). It is primarily secreted by activated macrophages during myocardial stress and stimulates fibroblasts to deposit collagen into the extracellular matrix (50), initiating the pro-fibrotic progression of the myocardium (51). Song et al. (52) and Yakar Tuluce et al. (53) found a connection between Gal-3 levels and the degree of LV hypertrophy in individuals with hypertrophic cardiomyopathy (HCM). Inhibition of the Gal-3 pathway, which is currently one of the most promising novels therapeutic targets for managing myocardial fibrosis, may prevent cardiac remodeling and dysfunction In the preclinical study (54). Furthermore, previous clinical research has shown that Gal-3 as a biomarker is correlated with CV disease risk stratification (55) or adverse prognosis (52, 56). In the field of HF, Gal-3 has proven to provide additive diagnostic and prognostic value (57, 58) and was recommended as a novel biological indicator for the risk stratification of HF at the 2013 American College of Cardiology Foundation (ACCF) /American Heart Association (AHA) (59). Therefore, an increasing amount of researchers have sought to elucidate the role of plasma Gal-3 in HFpEF through clinical trials in recent years. The majority of investigations focused on the relevance of plasma Gal-3 to the new-onset HFpEF or adverse outcomes of HFpEF patients. In contrast, others estimated the relationship of plasma Gal-3 with the pathological features of HFpEF, particularly cardiac remodeling or diastolic disorders (appraised by echocardiographic indices comprising LVMI, LAVI, E/A, E/e, and DT, et al.). There are, however, some inconsistencies in the findings of these investigations. Consequently, we carried out this meta-analysis to investigate the clinical significance of plasma Gal-3 in HFpEF.

The findings of the current meta-analysis illustrated a strong association between plasma Gal-3 and HFpEF clinical characteristics. First, high plasma Gal-3 levels were significantly associated with the increased risk of new-onset HFpEF (HR: 1.11; 95% CI: 1.04-1.19; *p* = 0.910, I^2^ = 0%; *P* = 0.002), implying that plasma Gal-3 can be applied as a biomarker to predict new-onset HFpEF. Second, increased plasma Gal-3 levels were correlated with the high risk of long-term adverse outcomes in HFpEF patients (all-cause death (HR: 1.55; 95% CI: 1.27-1.87; *p* = 0.138, I^2^ = 42%; *P* = 0.000) and the composite events [all-cause death and HF hospitalization (HR: 1.50; 95% CI: 1.30-1.74; *p* = 0.001, I^2^ = 61%; *P* = 0.000) or CV death and HF hospitalization (HR: 1.71; 95% CI: 1.51-1.94; *p* = 0.036, I^2^ = 58%; *P* = 0.000)], indicating that plasma Gal-3 can be utilized as a predictor of undesirable long-term prognosis in HFpEF patients. Finally, elevated Gal-3 levels were connected to E/e ratio (r: 0.425, 95% CI: 0.184-0.617; *p* = 0.000, I^2^ = 93%; *P* = 0.001) and DT (r: 0.502, 95% CI: 0.061-0.779; *p* = 0.001 I^2^ = 91%; *P* = 0.027), revealing that plasma Gal-3 can be applied as a predictor of LVDD in HFpEF populations.

### Strengths and Limitations

The strengths of our studies are as follows: Firstly, according to the NOS score, all of the included studies were of excellent quality, making our research findings more accurate and less prone to bias. Moreover, most of the selected studies employed multivariate analysis, reducing the influence of confounders. Thirdly, heterogeneity and sensitivity analyses were performed to identify the potential factors affecting the results and enhance the credibility of research findings. Nonetheless, some limitations need to be taken into consideration: First, the meta-analysis contained relatively few studies, limiting the ability to draw significant conclusions. Second, included studies involved various designs, and the baseline characteristics of the research differed, introducing bias and decreasing the reliability of our findings. Third, methods for detecting plasma Gal-3 varied in the included investigations, and the average plasma Gal-3 concentrations were variable, resulting in significant heterogeneity. Finally, publishing biases were confirmed using the Funnel plot, Begg's test, and Egger's test. As a result, our meta-analysis is an exploratory analysis, and additional high-quality original investigations are required to corroborate the findings of this study.

## Conclusion

In conclusion, plasma Gal-3 might be employed as an additional predictor for new-onset HFpEF, the adverse prognosis in HFpEF patients (all-cause death, the composite endpoints of all-cause death and HF hospitalization or CV death and HF hospitalization), and the severity of LVDD in HFpEF populations.

## Data Availability Statement

The original contributions presented in the study are included in the article/Supplementary Material, further inquiries can be directed to the corresponding author/s.

## Author Contributions

GD and JL designed this meta-analysis. GD reviewed the manuscript. YS performed the meta-analysis and wrote the manuscript. YS and XS developed the search strategy and performed literature searches and screening. Data extraction was conducted by WQin and WQia, while CL and CY evaluated the quality of the enrolled studies. All authors contributed to the article and approved the submitted version.

## Funding

This study was supported by grants from the National Natural Science Foundation of China (8207153216) and the Major Innovation Project of the China Academy of Traditional Chinese Medicine (CI2021A00903).

## Conflict of Interest

The authors declare that the research was conducted in the absence of any commercial or financial relationships that could be construed as a potential conflict of interest.

## Publisher's Note

All claims expressed in this article are solely those of the authors and do not necessarily represent those of their affiliated organizations, or those of the publisher, the editors and the reviewers. Any product that may be evaluated in this article, or claim that may be made by its manufacturer, is not guaranteed or endorsed by the publisher.
