# Supplementary material for: Clinical Implications of Plasma Galectin-3 in Heart Failure With Preserved Ejection Fraction: A Meta-Analysis
Source: Front Cardiovasc Med. 2022 Apr 14;9:854501. doi: 10.3389/fcvm.2022.854501 (PMC9046693; doi:10.3389/fcvm.2022.854501)
Supplement: Supplementary file 1 [file Data_Sheet_1.pdf]

# Supplemental materials

## 1. Supplementary Table 1: NOS score of the included studies

| Study | Quality indicators from the NOS | | | | | | | | Total scores |
|---|---|---|---|---|---|---|---|---|---|
| | selection | | | | comparability | Outcome | | | |
| | (i) | (ii) | (iii) | (iv) | (v) | (vi) | vii) | (viii) | |
| de Boer 2018(CHS) | ☆ | ☆ | ☆ | ☆ | ☆☆ | ☆ | ☆ | ☆ | 9 |
| de Boer 2018(FHS) | ☆ | ☆ | ☆ | ☆ | ☆☆ | ☆ | ☆ | ☆ | 9 |
| Bansal2019 | ☆ | ☆ | ☆ | ☆ | ☆☆ | ☆ | ☆ | | 8 |
| de Boer 2018 (PREVEND) | ☆ | ☆ | ☆ | ☆ | ☆☆ | ☆ | ☆ | | 8 |
| Beltrami2016 | ☆ | ☆ | ☆ | ☆ | ☆ | ☆ | ☆ | | **7** |
| Moliner2017 | ☆ | | ☆ | ☆ | ☆☆ | ☆ | ☆ | | 7 |
| Tromp2017 | ☆ | ☆ | ☆ | ☆ | ☆ | ☆ | ☆ | ☆ | 8 |
| Meijers2014(PRIDE) | ☆ | ☆ | ☆ | ☆ | ☆☆ | ☆ | ☆ | | 8 |
| Meijers2014 (UMD H-23258) | ☆ | ☆ | ☆ | ☆ | ☆☆ | ☆ | ☆ | | 8 |
| Edelmann2015 | ☆ | ☆ | ☆ | ☆ | ☆☆ | ☆ | ☆ | ☆ | 9 |
| Hage2017 | ☆ | ☆ | ☆ | ☆ | ☆ | | ☆ | ☆ | 7 |
| Chirinos2020 | ☆ | ☆ | ☆ | ☆ | ☆ | ☆ | ☆ | ☆ | 8 |
| Kanagala2020 | ☆ | ☆ | ☆ | ☆ | ☆ | ☆ | ☆ | ☆ | 8 |
| de Boer2011 | ☆ | ☆ | ☆ | ☆ | ☆☆ | ☆ | ☆ | | 8 |
| Carrasco-Sánchez2013 | ☆ | ☆ | ☆ | ☆ | ☆ | ☆ | ☆ | | 7 |
| French2016 | ☆ | ☆ | ☆ | ☆ | ☆ | ☆ | | ☆ | 7 |
| van der Velde 2013 (CORONA) | ☆ | ☆ | ☆ | ☆ | ☆ | ☆ | ☆ | ☆ | 8 |
| van der Velde 2013 (OACH) | ☆ | ☆ | ☆ | ☆ | ☆ | ☆ | ☆ | ☆ | 8 |
| Yamamoto2021 | ☆ | ☆ | ☆ | ☆ | ☆ | ☆ | | ☆ | 7 |
| Cui2018 | ☆ | ☆ | ☆ | ☆ | ☆☆ | ☆ | ☆ | | 8 |
| de Marco2021 | ☆ | ☆ | ☆ | ☆ | ☆☆ | ☆ | ☆ | | 8 |
| Trippel2021 | ☆ | ☆ | ☆ | ☆ | ☆☆ | ☆ | ☆ | | 8 |
| Mitic2020 | ☆ | ☆ | ☆ | ☆ | ☆ | ☆ | ☆ | | 7 |
| Wu2015 | ☆ | ☆ | ☆ | ☆ | ☆ | ☆ | | | 6 |
| Ansari2018 | ☆ | ☆ | ☆ | ☆ | ☆ | ☆ | ☆ | | 7 |
| Zile2016 | ☆ | ☆ | ☆ | ☆ | ☆ | ☆ | ☆ | | 7 |
| Polat2016 | ☆ | ☆ | ☆ | ☆ | ☆ | ☆ | | | 6 |

**The NOS evaluation included:** (i) representativeness of the exposed cohort, (ii) selection of the non-exposed cohort, (iii) ascertainment of exposure, (iv) demonstration that outcome of interest was not present at the start of the study, (v) comparability of cohorts based on study design or analysis, (vi) assessment of outcomes; (vii) was follow-up long enough for outcomes to occur, (viii) adequacy of follow-up of cohorts.

2. **Supplementary Figure 1: Funnel plot**

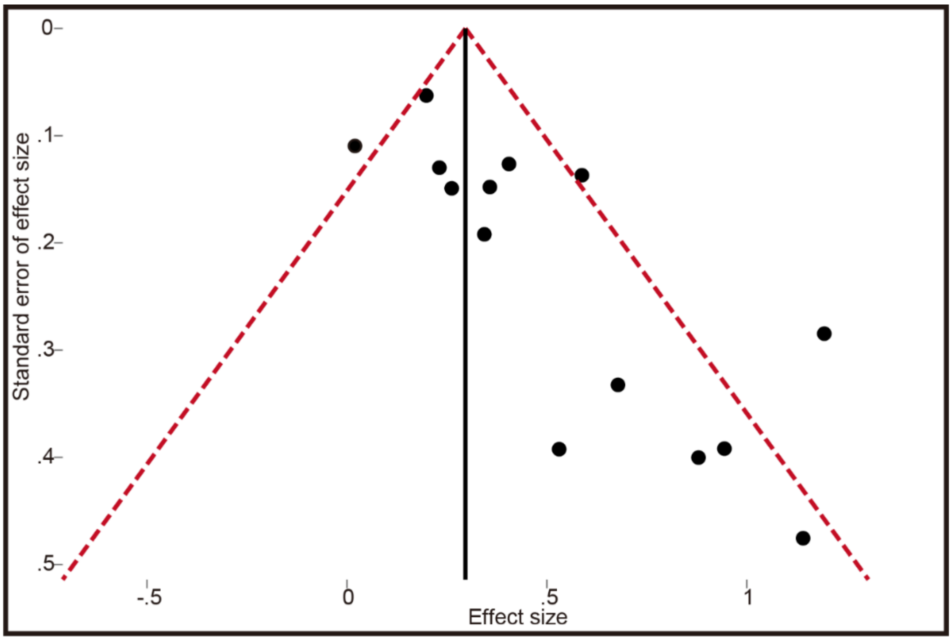


The Funnel plot of the meta-analysis of the relationship between high plasma Gal-3 levels and the composite outcomes of all-cause death and HF hospitalization was asymmetrical, indicating the existence of publication bias across included studies.

3. **Supplementary Figure 2: Begg's test**

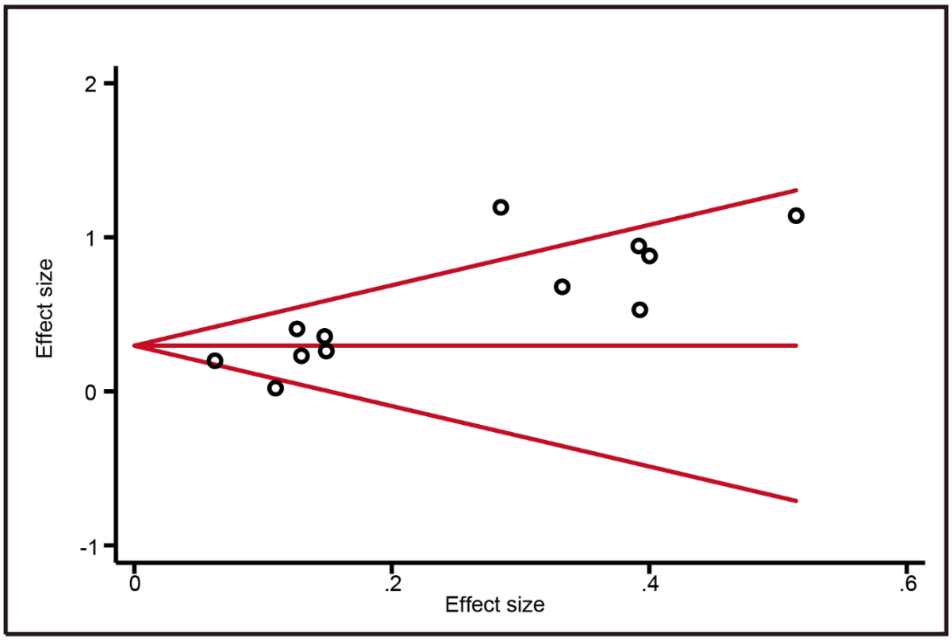


The p-value of Begg's test for the meta-analysis of the relationship between high plasma Gal-3 levels and the composite outcomes of all-cause death and HF hospitalization was 0.029, suggesting publication bias among the included studies.

4. **Supplementary Figure 3: Egger's test**

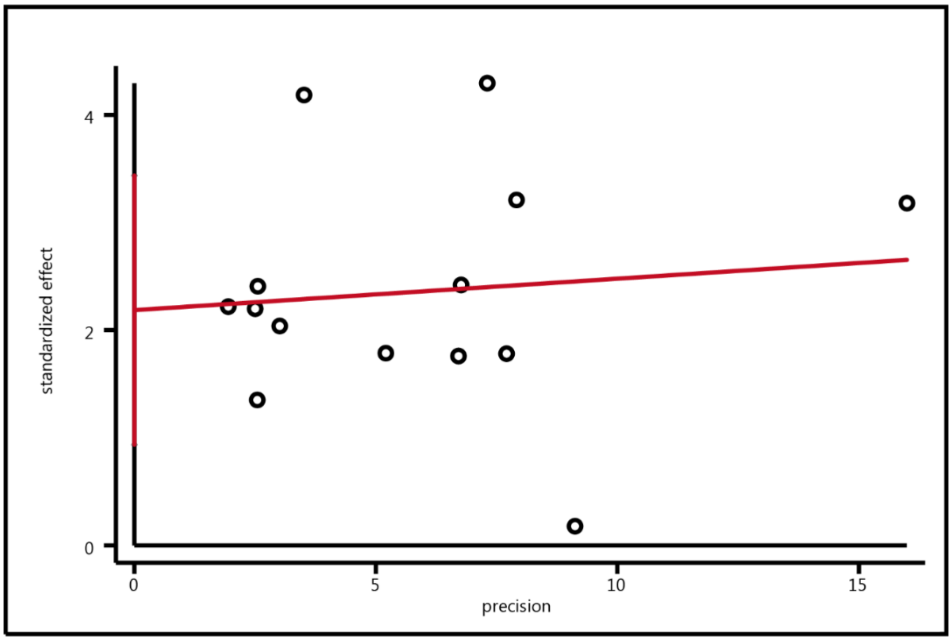


The p-value of Egger's test for the meta-analysis of the relationship between high plasma Gal-3 levels and the composite outcomes of all-cause death and HF hospitalization was 0.002, revealing publication bias in the included studies.
